# A Smooth Global Path Planning Method for Unmanned Surface Vehicles Using a Novel Combination of Rapidly Exploring Random Tree and Bézier Curves

**DOI:** 10.3390/s24248145

**Published:** 2024-12-20

**Authors:** Betül Z. Türkkol, Nihal Altuntaş, Sırma Çekirdek Yavuz

**Affiliations:** 1 Computer Engineering, Faculty of Electrical & Electronics, Yildiz Technical University, 34220 Istanbul, Türkiye; smyavuz@yildiz.edu.tr; 2 Computer Engineering, Faculty of Engineering and Architecture, Istanbul Gelisim University, 34310 Istanbul, Türkiye; naltuntas@gelisim.edu.tr

**Keywords:** RRT, Bézier Curve, USV, global path planning, path smoothing

## Abstract

Developing autonomous navigation techniques for surface vehicles remains an important research area, and accurate global path planning is essential. For mobile robots—particularly for Unmanned Surface Vehicles (USVs)—a key challenge is ensuring that sharp turns and sharp breaks are avoided. Therefore, global path planning must not only calculate the shortest path but also provide smoothness. Bézier Curves are one of the main methods used for smoothing paths in the literature. Some studies have focused on turns alone; however, continuous path smoothness across the entire trajectory enhances navigational quality. Contrary to similar studies, we applied Bézier Curves whose control polygon is defined by an RRT path and thus avoided a multi-objective formulation. In the final stage of our approach, we proposed a control point reduction method in order to decrease the time complexity without affecting the feasibility of the path. Our experimental results suggest significant improvements for multiple map sizes, in terms of path smoothness.

## 1. Introduction

Global path planning is one of the main research topics relating to robotic systems. Briefly, global path planning finds the optimal path from a starting point to a goal on a known map that is possibly occluded by static obstacles. The concept of optimality depends on the task, the robot and the environment properties. For example, a patrolling task necessitates maximum area coverage, whereas introducing less maneuverable robots to a scenario requires the use of smoother shapes. Therefore, path planning algorithms vary from those that search for the shortest path on a graph to those that perform the heuristic optimization of several constraints, such as average velocity, battery consumption, smoothness of the path, and so on. The field is dominated by three approaches and their improved versions [1]:

- Graph search-based algorithms such as Dijkstra’s Algorithm [2], A* [3] and their improvements [4,5,6].
- Sample-based search algorithms such as RPM [7], RRT [8] and RRT* [9].
- Heuristic optimization algorithms such as Genetic Algorithms [10] and Particle Swarm Optimization algorithms [11,12].

Sharp turns on the path may cause slower motion or even break behavior for robots, due to the safety of the system. The effects can vary significantly, ranging from changes in battery consumption to changes in travel time length. While most studies on smoothing global paths focus on path turns [13,14,15], obtaining smoothness for the whole path is also a topic studied in the literature [11,12,16].

Sample-based algorithms search for the optimal path through samples of the map instead of checking every grid cell. Although they do not guarantee the absence of sharp turns in the path, they remain popular due to their ease of implementation, computational speed and high performance in complex environments. Generally, instead of optimizing the smoothness of the path within the path planning procedure, an individual smoothing procedure is performed after path planning. For instance, an RRT path planner was followed by a Quadratic Bézier Curve-based smoothing procedure in [17]. Similarly, an improved version of the RRT* algorithm followed by a B-Spline interpolation smoothing procedure was proposed in [18].

Bézier Curves are one of the common smoothing approaches that are independent from the path planning method [5,13,16]. The main problems with using Bézier Curves relate to the feasibility of the path and the complexity of the curve generation, which depends on the number of control points. As a solution for this problem, lower-degree Bézier Curves, such as Cubic Bézier Curves [16] or Quadratic Bézier Curves [12], were preferred in some studies. However, the authors of [11] showed that higher-order curves provide continuity and, therefore, more natural turns in the path and fewer sharp breaks during travel. The feasibility of the path depends on the locations of the control points and, thus, in the literature, optimization algorithms were used to locate control points [10,12,19] that could create feasible Bézier Curves in terms of obstacle avoidance and smoothness of shape, including higher-order curves. The disadvantage of such optimization methods is their computational overhead.

To the contrary, in this study, we propose a method that uses an RRT global path, which already avoids obstacles, as the control polygon to create Bézier Curves that benefit from their variation-diminishing property. In addition, simulation experiments on four different maps show that the method provides an improvement in total travel time and reduces the percentage of sharp breaks.

The remainder of this paper is structured as follows: In Section 2, we further elaborate on the RRT and Bézier Curve methods; Section 3 provides the details of the proposed method; and the experimental results are provided in Section 4.

## 2. Background

### 2.1. RRT

The RRT is a sample-based searching algorithm which has been used for path planning since 1998 [8]. In a search space, represented by an occupancy map, it expands a tree using an initial point as the first node. Then, a random sample of an obstacle-free space on the map is chosen and the closest node of the tree is selected. If there is no obstacle between the node and the point, a new node is created with a distance of the given step size to the node, and the process continues for each node of the tree until either the goal is included in the tree or a maximum number of iterations is reached. The pseudo-code of the RRT-based global path planning algorithm is shown in Table 1, with the initial point of the algorithm being the start position of the robot. It is concluded that the goal is reached if a new node is added to the RRT within a given goal tolerance. In our experiments, we set the step size parameter d to 0.2 m, the goal tolerance to 0.5 m and the maximum number of iterations to 40,000.

### 2.2. Bézier Curves

Bézier Curves are parametric curves that were originally invented by Pierre Bézier in order to design the shapes of Renault cars in the 1970s [20]. At present, computer graphics, animations and robotics are the main application areas of these curves. In path planning, they are primarily used for creating smooth, traceable path for robots, creating a smooth connection between arbitrary start and end points with respect to the given discrete control points. The number of control points varies from 0 to n, where the parameter n is application-specific. The main properties of Bézier Curves are as follows:

- End Point Interpolation: This property provides a curve that passes through the control polygon end points, forming a complete path between the initial point of the robot and the target point.
- Variation Diminishing: The curve cannot fluctuate beyond the boundaries of the control polygon. As we used the RRT global path as the control polygon for the curve, obstacle avoidance was achieved through the combined effect of this property and the RRT algorithm.

In [21], it was discovered that Bézier Curves can be approximated by Bernstein polynomials (i.e., in the general form of the curves, control points are defined by Bernstein polynomial coefficients). Therefore, we applied these findings and used Bernstein polynomials to implement the Bézier Curve generation. The pseudo-code of our iterative Bézier Curve generation algorithm is provided in Table 2.

## 3. Method and Motivation

### 3.1. Motivation

USVs performing patrol duties are increasingly utilized to ensure surface security, including in shipyard-like facilities along the coastline. Thus, we were motivated to create such a security system around a maze-shaped shipyard.

Similar to other mobile robotic tasks, global path planning is the first step of the navigation in our application. The RRT remains one of the most widely used global path planning algorithms and continues to be improved upon [22,23,24]. The advantage of this algorithm is its rapid calculation, while its disadvantage is that determining a path using random points may cause poor optimization. However, this disadvantage can be tolerated when the environment needs re-planning due to dynamic factors.

As our motivation was to create a system for patrolling around an active shipyard, the RRT best suited the mission. However, the RRT does not guarantee smooth connections between nodes and thus may cause more sharp turns and more sharp breaks. As the number of motion commands required to track the global path increases, the more break commands the code contains and the longer it takes to reach the goal. In [17], RRT node connections were refined to simplify the path; however, an additional procedure was used to ensure smooth turns. In contrast to that work, we obtained a smooth RRT using a single refinement. Smoothing the whole path, instead of just a part of it, provides continuity and a naturally smooth path [11].

### 3.2. Proposed Method

Our method can be described briefly as comprising three main parts: classical RRT [8], control point extraction using two different approaches and Bézier Curve generation [25].

**Figure 1:**
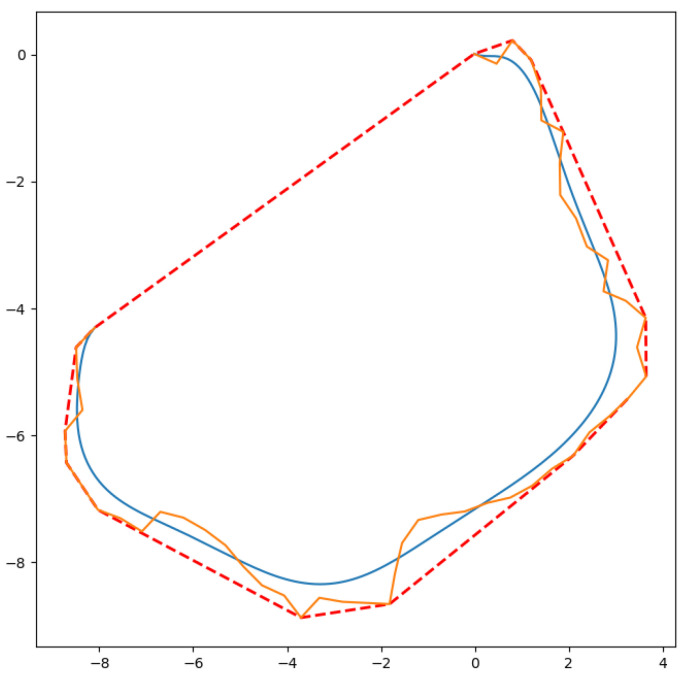
A Bézier Curve (blue curve), its control polygon (orange polygon) and its convex hull (red dashes).

A Bézier Curve cannot overflow the convex hull that is formed by its control polygon due to its variation-diminishing property. In Figure 1, a control polygon of a Bézier Curve (orange polygon), the curve (blue curve) produced by it and the convex hull of the same Bézier Curve (red dashes) are illustrated. In our case, the order of the points is important for generation of the curve as a traceable path because the control polygon is defined as the connections between the control points, where the RRT nodes are the control points and the connections are the RRT path. In other words, the control polygon is the RRT path itself. Therefore, the proposed method produces a curve bounded by an RRT global path that avoids obstacles yet is able to smooth turns.

### 3.3. Control Point Reduction

**Figure 2:**
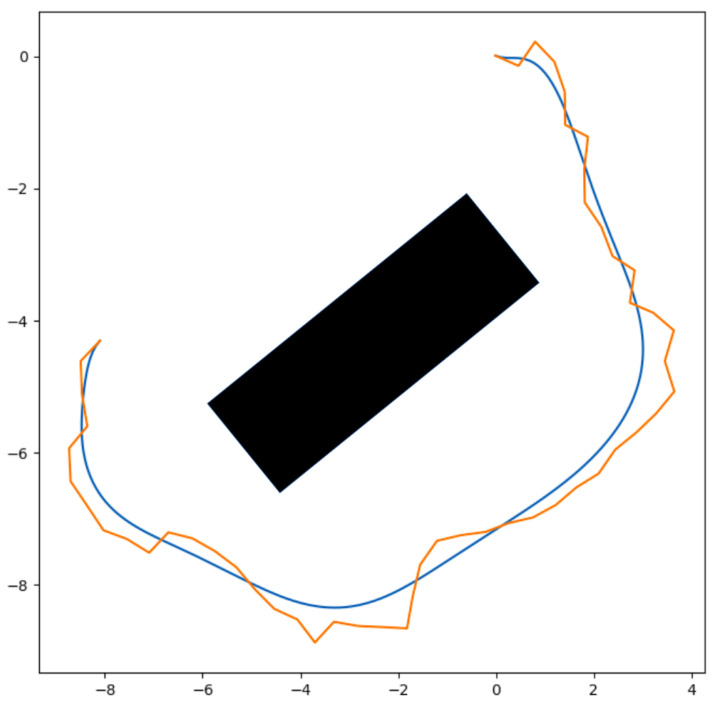
An RRT path (orange polygon) avoiding the obstacle (black rectangular) and the Bézier Curve defined by that polygon (blue curve).

In the first control point extraction method, the *x* and *y* coordinates of nodes on the map are used as 2D control points for the Bézier Curve. Although a traceable smooth path can be generated with this information alone, because of the complexity of the Bézier Curve generation, an additional point selection step is required in order to reduce the number of control points. The creation of a feasible path that avoids obstacles is ensured by containing every node in the control polygon, which ensures that the curve is bounded by a narrow field around the path. This field is bounded by the most distant point of the plane formed by the RRT path, which is also the control polygon, as mentioned before (see Figure 2).

**Figure 3:**
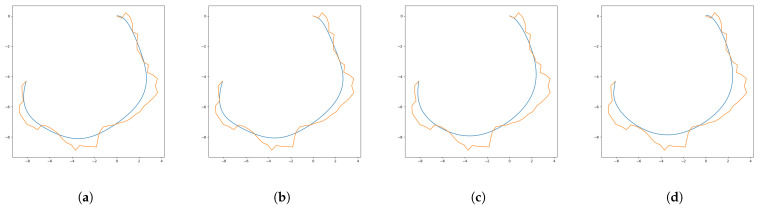
RRT path (orange polygon) as original control polygon and Bézier Curve (blue curve) after filters: (**a**) 3 × 1 mean filter, (**b**) 3 × 1 median filter, (**c**) 5 × 1 mean filter, (**d**) 5 × 1 median filter.

Control point reduction counters the complexity of the Bézier Curve generation. Hence, in this study, two sizes of two different types of filters were applied individually to the original control points to reduce their number. In Figure 3a–d, the Bézier Curves after the control point reduction procedure using the 3× 1 mean filter, 3 × 1 median filter, 5 × 1 mean filter and 5 × 1 median filter are illustrated, respectively. The blue curves are the curves with the filtered number of control points and the orange polygons are the original control polygons created by the RRT nodes. It is obvious from Figure 3 that point reduction provides a smoother-shaped path. However, it is also further from the original path, which might end at an unfeasible path. Therefore, filters with wider sizes were not considered for our application.

As we applied filtering to the control points, we risked ending up with an inconsistent set of control points. To analyze whether such a case might happen, we double-checked whether our trajectories overlapped with the occupied regions of the costmap in an exhaustive manner. In this way, we experimentally verified that, after control point reduction, all of our obtained trajectories were still in the feasible regions.

As the second control point extraction method, we applied a 3 × 1 filter as follows: On the calculated RRT global path, we grouped every three nodes on the path in ascending order by *x* coordinate. Then, we selected the medians of the groups as control points. This reduction in the number of control points helps reduce the complexity, but does not cause a risk of information loss. Hence, the stride of the sequences of three nodes was considered to be 2. We also applied a mean filter to the same group of nodes, in which we selected the means of the three nodes as control points.

Assume *n* nodes of a global path $N_{0},N_{1},N_{2},\ldots ,N_{n-1}$ which are also control points for an (n−1)-degree Bézier Curve $P_{0},P_{1},P_{2},\ldots ,P_{n-1}$ where $P_{0}=N_{0}$ and $P_{n-1}=N_{n-1}$ are the start and end point of the curve, respectively. The *j*th control point $P_{j}$ is calculated as shown in Equation (1):

(1) $$\begin{array}{cc} P_{j} & =Filter\left(N_{i-1},N_{i},N_{i+1}\right)\mathrm{where}\\ & j=\left\{1,2,3,\ldots ,\left\lfloor \frac{n-1}{2}\right\rfloor \right\},i=2k+1\mathrm{and}k\in Z_{\geq 0} \end{array}$$

Filter is a function that calculates the mean or median of the parameters. We tested the above filters individually, and the experimental design and test results are discussed in detail in Section 4.

In the third control point extraction method, we aimed to further reduce the number of control points via applying a method similar to the previous one in which the filter size is expanded to 5 × 1 so that the median and mean filters are applied to five nodes to extract each control point of the curve. In this case, the stride of the sequences of five nodes was considered to be 3, so we reduced the number of points even further and still avoided losing too much of the information coming from the RRT. Assuming the same nodes above the *j*th control point, $P_{j}$ is shown in Equation (2):

(2) $$\begin{array}{cc} P_{j} & =Filter\left(N_{i-2},N_{i-1},N_{i},N_{i+1},N_{i+2}\right)\mathrm{where}\\ & j=\left\{1,2,3,\ldots ,\left\lfloor \frac{n-2}{3}\right\rfloor \right\};i=2+3\left(k-1\right)\mathrm{and}j,i,k\in Z_{\geq 1}. \end{array}$$

Table 3 shows the pseudo-codes of the 3 × 1 and 5 × 1 mean filter procedures, while Table 4 shows the pseudo-codes of the 3 × 1 and 5 × 1 median filter procedures.

### 3.4. Bézier Curve Generation

Assume a degree of the n-1 Bézier Curve B(t) is generated over *n*control points $P_{0},P_{1},\ldots P_{n-1}$ where the parameter of the curve t∈[0,1]. In the following way, Bernstein polynomials can be used in the Bézier Curve generation to calculate the coefficients (i.e., weights) of the control points, which ensures that the start and end points of the curve are the first and last control points, respectively. A Bernstein polynomial gives a weight of 1 to a control point when t=0, which means the first control point $P_{0}$ is the starting point of the curve. Similarly, it gives a 0 weight to a control point when t=1, where control point $P_{n-1}$ is the last point of the curve. When 0<t<1, control points within $P_{1}$ and $P_{n-2}$ are given weights where the sum of them defines the points on the Bézier Curve. A Bézier Curve whose coefficients are defined by Bernstein polynomials can be expressed using Equation (3):

(3) $$B\left(t\right)=\Sigma \left(B_{\left\{i,n\right\}}\left(t\right)\cdot P_{i}\right)=\Sigma \left(C\left(n,i\right)\cdot \left(1-t\right)\left(n-i\right)\cdot t_{i}\cdot P_{i}\right)$$

where $P_{i}$ are the control points, and $B_{\left\{i,n\right\}}\left(t\right)$ are the Bernstein polynomials [25]. In our case, for an RRT path with n+1 nodes, we generated Bézier Curves with three different degrees: *n*, $\left\lfloor \frac{n-1}{2}\right\rfloor$ and $\left\lfloor \frac{n-2}{3}\right\rfloor$. The points extracted via the control point extraction method were used as the control points of the curves, and the parameter *t* was calculated iteratively as shown in Equation (4):

(4) $$\begin{array}{cc} t & =\Delta \cdot j\mathrm{where}\\ & \Delta =\frac{1}{n-1}\mathrm{and}j=\left\{0,1,\ldots ,n-1\right\} \end{array}$$

### 3.5. Time Complexity of the Method

The iterative implementation of the RRT has a time complexity of $O(n^{2})$ where n is the number of nodes. In the first control point extraction method, the number of control points is *n* and we used all nodes directly. After the control point reduction procedures, the number of control points can be expressed as $\left\lfloor \frac{n-1}{2}\right\rfloor$ and $\left\lfloor \frac{n-2}{3}\right\rfloor$, respectively. We iteratively implemented the Bézier Curve expression above, which has a time complexity of $O(n^{3})$. In all three cases, when adding the RRT part of the algorithm, the total time complexity was $O\left(n^{2}\right)+O\left(n^{3}\right)$, which can also be expressed as $O(n^{3})$. Although the time complexity was increased, the total runtime of the algorithm in the simulation tests kept decreasing. As is mentioned in Section 4, this interesting result was an effect of the smoothness of the path and the shortcuts around the sharp turns. The smoothness also had an effect on linear motion commands. Sharp turns cause sharp breaks, which can affect the performance of the USV in terms of travel time. We provided an improvement in travel time with a procedure that can be implemented and tested easily.

### 3.6. Experimental Environment on Simulator

The tests were performed in the Gazebo 9.19 simulation environment and ROS Melodic on Ubuntu 18.04. The Gazebo environment is quite flexible, offers a variety of ready models for the user and allows fast customization of these models. It also provides simulator performance validation with the Real-Time Factor, which shows simulation speed over system time, which was between 0.98 and 1.0 during the tests, where 1.0 means the simulation runs in real time. For generality, we used an existing open-source USV model which was developed in the context of autonomous online path planning and path-following control of USVs [26]. This model provides a novel robot model and an environment model where the environment is an obstacle-free ocean world. In contrast to flat-ground terrains, the robot is allowed to ascend and descend using the z coordinate of the sea. The model USV has a footprint of 200 × 105 × 85 cm and two propellors at the rear, and is capable of reaching a maximum speed of 1.4 m/s. It is equipped with 2D-LIDAR, IMU and GPS sensors, which we concluded were realistic enough for our simulation scenario.

In our experiments, we generated obstacles around the USV to create different types of maps. All the obstacles we generated were rigid, and it was strictly forbidden for the robot to make contact with them. The main steps of our approach, the RRT, control point extraction and Bézier Curve generation procedures, were implemented using C_++_ on ROS Melodic.

### 3.7. Tests

**Figure 4:**
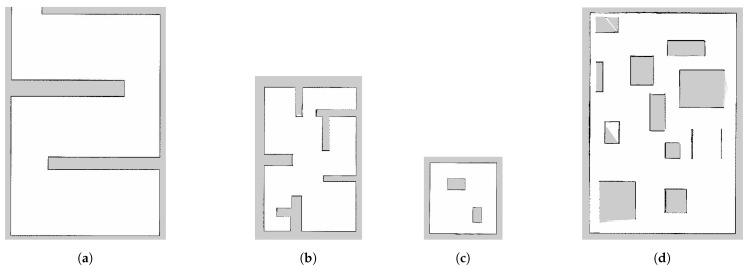
Different maps used for testing: (**a**) Map1, (**b**) Map2, (**c**) Map3, (**d**) Map4.

The method was tested on four different 2D maps: Map1, Map2, Map3 and Map4. Map1 had an area of 620.2 m^2^ with obstacles placed to obtain the shape of a maze-shaped road and the occupied space took 9.4% of the whole area; Map2 had an area of 383.8 m^2^ with a larger occupied space covering around 16%; Map3 was 182 m^2^ and had only two static obstacles that took 7% of the whole area; Map4 was also 620.2 m^2^ and had the largest occupied area covering 25%. Figure 4a–d illustrate Map1, Map2, Map3 and Map4, respectively. The maps were obtained using the Gmapping package on ROS [27], a well-known SLAM method involving a particle-filter-based algorithm that was proposed in 2007 [28]. Twenty-four tests were performed on each of the maps in the following path planning cases:

- Classical RRT;
- Bézier Curve without point reduction (BC);
- Bézier Curve with point reduction by 3 × 1 mean filter (Mean3);
- Bézier Curve with point reduction by 3 × 1 median filter (Median3);
- Bézier Curve with point reduction by 5 × 1 mean filter (Mean5);
- Bézier Curve with point reduction by 5 × 1 median filter (Median5).

## 4. Experimental Results and Discussion

The proposed method was divided into six parts and each part was tested on four different maps. The parts of the method differed from each other in terms of the path planning algorithm and control point extraction method, while the maps differed from each other in size and environment complexity. In total, 240 separate tests were performed for 24 map–case combinations. Each of the combinations was tested 10 times and their averages were considered as the results. The results are tabulated in Table 5.

Before discussion of the results, we would like to recall that the time complexity of the iterative implementation for the RRT is $O(n^{2})$ and the time complexity of the iterative implementation of the Bézier Curve is $O(n^{3})$, where n is the number of nodes and number of control points, respectively. Briefly, the results show that, even when the time complexity of the path planning algorithm was increased, the total time to reach the goal decreased dramatically for the different types of maps covering an area of up to about 620 m^2^.

**Figure 5:**
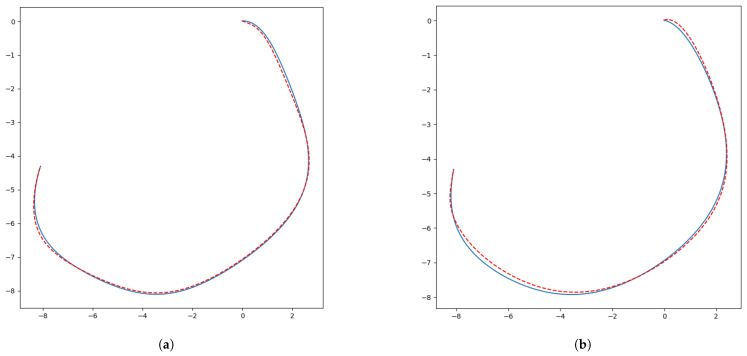
Different filter types resulted in similar curves: (**a**) 3 × 1 mean filter (blue curve) and 3 × 1 median filter (red dashed curve), (**b**) 5 × 1 mean filter (blue curve) and 5 × 1 median filter (red dashed curve).

The average improvement in the total simulation time was 20.92%, with the best improvement of 38.3% being achieved for Map4 and the least improved result for Map3, with an average of 9.7%. Improvement was not affected by filter type; instead, performance depended on the number of control points and the shape of the curve. In Figure 5a, the blue curve shows the curve created by the 3 × 1 mean filter control points, while the red dashes show the curve created by the 3 × 1 median filter control points. Similarly, in Figure 5b, the blue curve is the 5 × 1 mean filter curve and the red dashes delineate the 5 × 1 median filter one. It can be observed that the two types of filters give very similar curves. Control point reduction saves the path from useless fluctuation and thus gives the robot fewer breaks; therefore, the filtered path cases were quicker than the RRT paths and even quicker than the original Bézier Curve paths.

**Figure 6:**
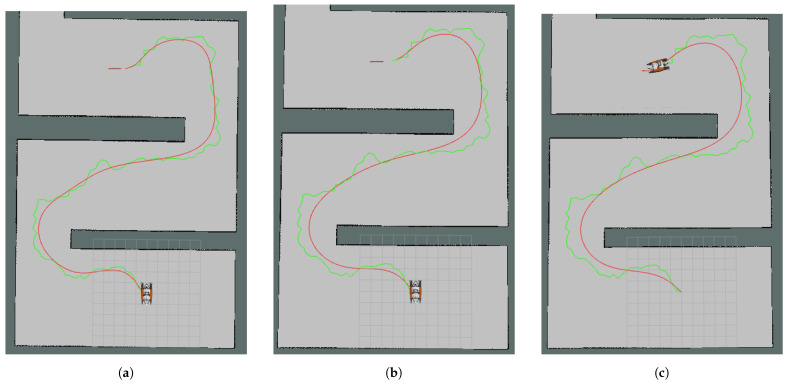
Curves (red curves) obtained for Map1 using the same control polygon which is also an RRT path (green polygon): (**a**) without control point reduction, (**b**) with 3 × 1 median filter control point reduction, (**c**) with 5 × 1 median filter control point reduction.

In Figure 6a–c, path examples are shown for Map1. The red curves are the Bézier Curve paths and the green polygon is the original RRT whose nodes were used to create curves. Although the 5 × 1 filtered paths were feasible in every test, more information loss could be a disadvantage; therefore, widening the filters was not considered. Since the RRT was the control polygon for the original Bézier Curve path, the variation-diminishing property could be observed on the path (see Figure 6a). Even though it reduces the execution time less than other control point extraction approaches, it might be safer to use the unfiltered curve in more complex environments.

**Figure 7:**
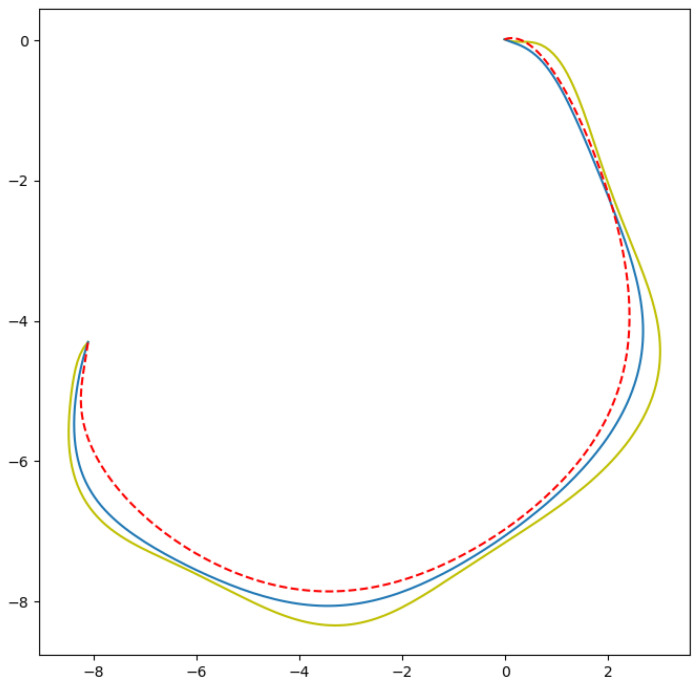
Difference in shape of curve with n control points (yellow curve), with $\left\lfloor \frac{n-1}{2}\right\rfloor$ control points (blue curve), with $\left\lfloor \frac{n-2}{3}\right\rfloor$ control points (red dashed curve).

In Figure 7, the curves created by the original control polygon and the 3 × 1 filtered and 5 × 1 filtered control polygons are illustrated. A change in the shape of the curve can be observed where the yellow curve has the original number of control points, the blue curve is the 3 × 1 filtered control points and the red dashes are the 5 × 1 filtered control points.

### 4.1. Results for Map1

The longest runtime for the Bézier Curve generation was 29.1 ms, seen for the largest map, Map1, with 121 control points. After control point reduction, the runtime was decreased to 4 ms. The main disadvantage of Bézier Curves is that the runtime increases rapidly with the number of control points. However, to create a feasible path, avoiding obstacles is only possible when the information from the original RRT nodes is kept. Total simulation time to reach the goal decreased from 463.19 s to 409.3 s for the slowest algorithm and to 380.98 s for the quickest algorithm for Map1. This result was achieved due to smoother turns and fewer sharp break commands, caused by the smoother path. Tracking the original RRT caused two more sharp breaks than the non-filtered Bézier Curve path and fifteen more than the 5 × 1 filtered one. In other words, a smoother path creates fewer sharp break commands to be sent to the robot. There was around a 16% time improvement for this map. In Figure 6, path examples for Map1 are shown where Figure 6a is the curve of the original control points, Figure 6b is the curve of the 3 × 1 median filter control points and Figure 6c is the curve of the 5 × 1 median filter control points.

### 4.2. Results for Map2

**Figure 8:**
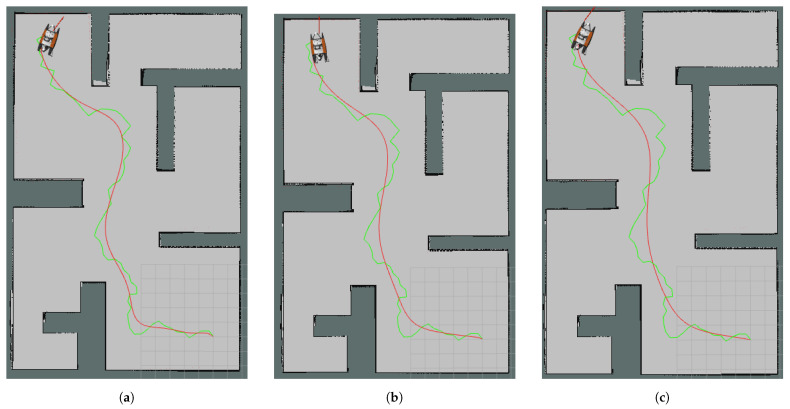
Curves (red curves) obtained for Map2 using the same control polygon which is also an RRT path (green polygon): (**a**) without control point reduction, (**b**) with 3 × 1 mean filter control point reduction, (**c**) with 5 × 1 mean filter control point reduction.

Map2 was around 380 m^2^ and had more occupied area than the other two maps. In this case, the 74-node original RRT was calculated as the path and control polygon of the Bézier Curve. The best improvement in simulation time on this map was again achieved by the path with the 5 × 1 median filter applied, reaching 25.9%. A dramatic decrease in time occurred at about 1.25 min and the USV reached the goal in about 5 min in the case with the worst simulation time. Furthermore, the percentage of sharp break commands sent to the robot decreased from 41‰ to 4‰. In Figure 8a–c, the curves of the original control points, the 3 × 1 mean filter control points and the 5 × 1 mean filter control points for Map2 are shown, respectively.

### 4.3. Results for Map3

**Figure 9:**
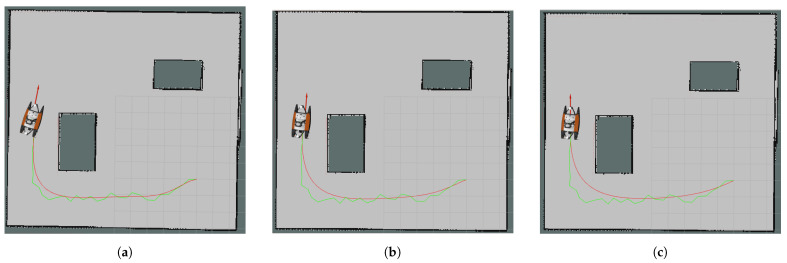
Curves (red curves) obtained for Map3 using the same control polygon which is also an RRT path (green polygon): (**a**) without control point reduction, (**b**) with 3 × 1 mean filter control point reduction, (**c**) with 5 × 1 mean filter control point reduction.

Map3 had the smallest area and the least complex environment. Even in this simple case, the number of sharp breaks and total simulation time were decreased, with the total improvement reaching about 11%. Due to the small size of the area, this case had the fewest control points of all the test cases, and the path planning procedure was almost as quick as the original RRT. In Figure 9, Figure 9a is the curve of the original control points, Figure 9b is the curve of the 3 × 1 mean filter control points and Figure 9c is the curve of the 5 × 1 mean filter control points.

### 4.4. Results for Map4

In order to demonstrate the performance of our method when it is applied to a challenging task, we conducted experiments on Map4. This map represents a real-world scenario where the shape and size of the obstacles vary significantly. The tiny obstacles represent buoys, while larger obstacles represent islands. The area coverage was 620 m^2^ and, in contrast to the previous maps, the occupied areas covered 25% of the total area. To observe the trajectory changes in a controlled manner, we performed two sets of experiments. In the first experiment, the map was only populated with islands. In this case, the navigation was easier as there were no buoys. In the second experiment, buoys were introduced as well as islands, which added another level of complexity.

**Figure 10:**
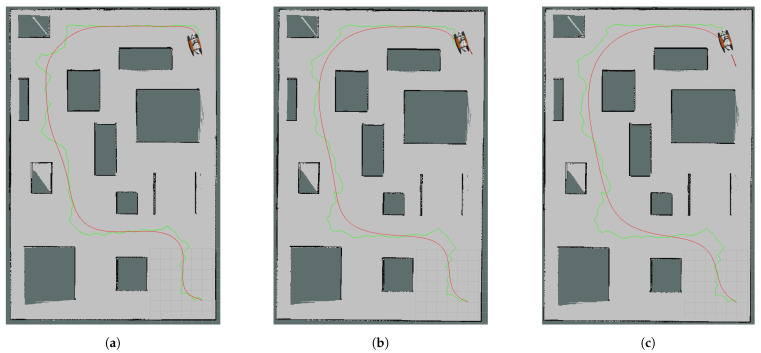
Curves (red curves) obtained for Map4 using the same control polygon which is also an RRT path (green polygon): (**a**) without control point reduction, (**b**) with 3 × 1 mean filter control point reduction, (**c**) with 5 × 1 mean filter control point reduction.

**Figure 11:**
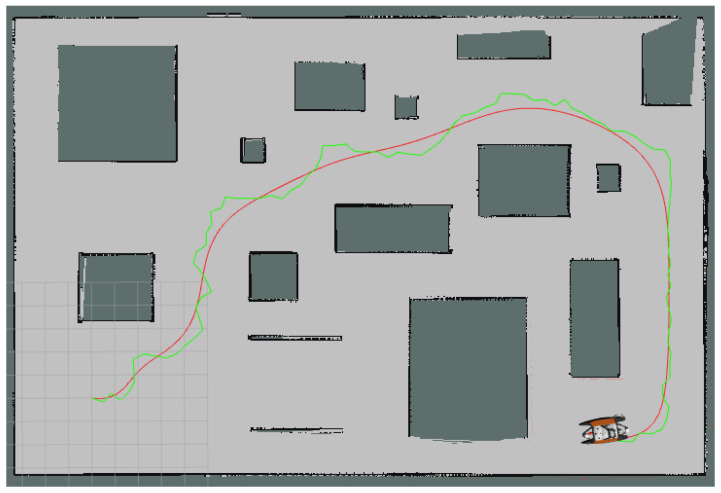
The Bézier Curve (red curve) and its control polygon (green polygon) for the second Map4 scenario.

In Figure 10, we depict the results for the first Map4 scenario. With and without control point reduction, we consistently obtained smooth solutions. Figure 11 depicts the results for the second scenario. In this experimental setting, we noticed that it was quite a lot harder for the RRT to find the initial control points, due to the highly constrained nature of the problem.

**Figure 12:**
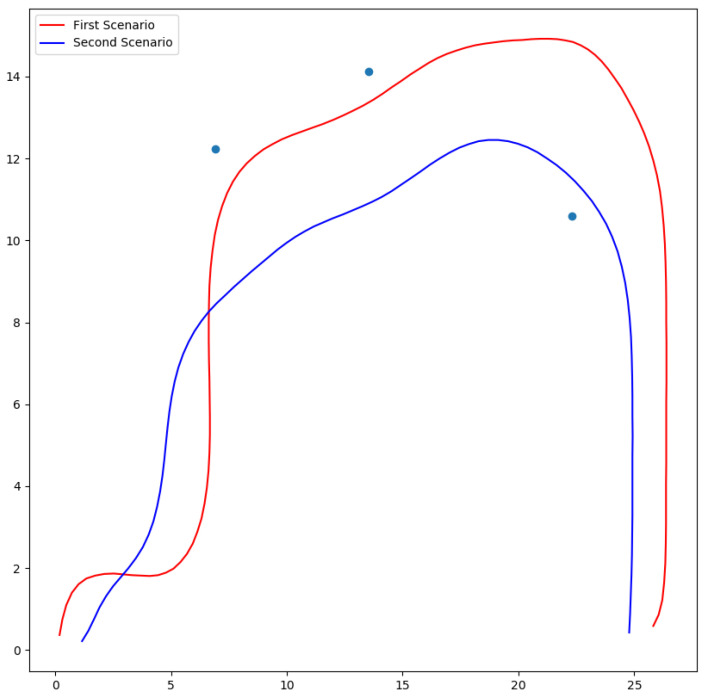
The paths created by the proposed method for Map4 without buoys in the first scenario (red curve) and Map4 with buoys in the second scenario (blue curve). Buoys are represented by the blue dots.

For clarity, we overlap the trajectories from the first and second scenarios in Figure 12. The red curve is our solution for the first scenario, the case with multiple islands and no buoys; the blue curve is the final solution in the case with constraints imposed by islands and buoys. We observed that the USV had difficulty following the original RRT path because of the sharp turns, and a re-planning procedure was performed at least once by the navigation package. The best runtime for path planning was 3.8 ms, achieved by the Mean5 filter, and the worst was 28.6 ms, for the original BC planning without filtering. As expected, the RRT had an average performance time of 0.23 ms. However, the total simulation time of robot travel from the start point to the goal when following the RRT path was 571.6 s, while the best result was 352.2 s, achieved with Median5 filtering, which is equal to a 38.3% improvement.

Table 5 shows the simulation results for the maps detailed above, and an explanation of the columns is provided below:

- First column (Test): Name of the test with the format “mapName_testNo”.
- Second column (Algorithm): The global path planning algorithm, where RRT and BC represent the classical RRT and the proposed method, respectively.
- Third column (Number of Points/Nodes): Number of control points used to generate the Bézier Curve/number of nodes in the path generated by the RRT.
- Fourth column (Point Reduction Type): Control point reduction method with the format “filterName-filterSize”. Filter types and size information are provided in Section 3.
- Fifth column (Algorithm Runtime (ms)): Time elapsed between when the goal is assigned and when the robot starts moving in milliseconds.
- Sixth column (Travel Runtime (s)): Time elapsed between when the robot starts moving and when the robot reaches the goal in seconds.
- Seventh column (cmd_vel ‰ of Sharp Breaks Over All Commands): Percentage of sharp breaks over all commands. It was calculated via analyzing the motion commands published by the cmd_vel topic in ROS. If a linear motion command was decreased by 25% compared to the previous one, it was defined as a “sharp break”. For example, in the test named MAP1_1, thirty-three linear motion commands were defined as sharp breaks out of thousands of commands published.
- Eighth column (Improvement in Time Compared to RRT (%)): The percentage decrease in total travel time compared to that of the classical RRT.

## 5. Conclusions

Smoothing the global path provides safer and faster travel for mobile robots, and is particularly crucial in settings where USVs have poor maneuverability. To address such settings, we proposed a novel global path planner that is a combination of the RRT and higher-order Bézier Curves. Similar studies have a tendency to avoid using higher-order Bézier Curves due to their time complexity and generate them through formulating a multi-constraint optimization problem. However, we have shown that, using RRT as a control polygon for a Bézier Curve, it is possible to obtain a feasible, smooth path via further reduction. All of our experimental tests showed promising results for both control point extraction approaches, in terms of movement feasibility and travel time reduction. Briefly, our main contributions and findings are summarized below:

- Although using higher-order Bézier Curve generation takes slightly more computation time, the resulting smooth trajectory causes a significant reduction in the total travel time of the robot.
- It was shown that the obstacle avoidance property of RRT does not vanish when it is defined as the control polygon of the Bézier Curve.
- Tests have shown that more information from the RRT provides safer paths. Another level of improvement can be achieved via eliminating unnecessary nodes. However, path feasibility must be proven in order for it to remain a safe distance from the obstacles.

In our future work, we aim to further extend this work in several distinct directions. First, we aim to analyze how our curve generation is influenced when we base the process on more advanced path planning techniques, such as RRT*. Second, although the experimental findings of this current work verify that Bézier Curves can successfully avoid obstacles, we are still interested in finding a satisfactory theoretical explanation as to why Bézier Curves can avoid obstacles when fewer control points are used. Finally, we are interested in expanding this work to even more complicated scenarios involving moving objects and obstacles.

## Figures and Tables

**Table 1:** RRT-based global path planning algorithm.

Algorithm RRT
Input: Number of nodes *K*, step size *d*, startPose *S*, finalPose *F*, goalTolarance *G*
Output: path RRT
RRT ← initializePath(*S*)
for k=1 to *K* do
n_rand ← randomPoint (searchSpace)
n_near ← getNearestNeighbor (n_rand, RRT)
if no obstacle between n_rand and n_near do
n_new ← extendTree(n_rand, n_near, *d*)
RRT ← addToPath (n_new)
if distance(*F*, n_new) ≤ *G*
Success Message and Break
return RRT

**Table 2:** Bézier Curve generation algorithm based on Bernstein polynomials.

Algorithm BC
Input: Number of control point *N*, N×2 matrix containing pairs of *x*, *y* coordinates of *N* points
Output: path BC
BC ← initializePath($x_{0}$,$y_{0}$)
*t* ← 0
result ← 0
for j=0 to *N* do
$t=\frac{1}{N-1}\cdot j$
result=result+ outerProduct(Bernstein (*j*,N−1,*t*), points(*j*))
BC ← createPath(result)
return BC

**Table 3:** Control point reduction via mean filter algorithm.

Algorithm: meanFilter3
Input: RRT (N×2 matrix containing pairs of *x*, *y* coordinates of *N* points)
Output: newPoints (2D vector containing *x*,*y* coordinates of extracted control points)
newPoints ← initializeVector()
*i* ← 1
Loop while i<N−1 do
*X* = mean (x.RRT(i−1), x.RRT(*i*), x.RRT(i+1))
*Y* = mean (y.RRT(i−1), y.RRT(*i*), y.RRT(i+1))
Add (*X*, *Y*) at the end of newPoints vector
Increment *i* by 2
End Loop
return newPoints
**Algorithm: meanFilter5**
Input: RRT (N×2 matrix containing pairs of *x*, *y* coordinates of *N* points)
Output: newPoints (2D vector containing *x*,*y* coordinates of extracted control points)
newPoints ← initializeVector()
*i* ← 2
Loop while i<N−2 do
*X* = mean (x.RRT(i−2), x.RRT(i−1), x.RRT(*i*), x.RRT(i+1), x.RRT(i+2))
*Y* = mean (y.RRT(i−2), y.RRT(i−1), y.RRT(*i*), y.RRT(i+1), y.RRT(i+1))
Add (*X*, *Y*) at the end of newPoints vector
Increment *i* by 3
End Loop
return newPoints

**Table 4:** Control point reduction via median filter algorithm.

Algorithm: medianFilter3
Input: RRT (N×2 matrix containing pairs of *x*, *y* coordinates of *N* points)
Output: newPoints (2D vector containing *x*,*y* coordinates of extracted control points)
newPoints ← initializeVector()
localArray: 3 × 2 array containing *x*,*y* coordinates to be sorted
*i* ← 1
Loop while i<N−1 do
localArray ← (RRT(i−1), RRT(*i*), RRT(i+1))
sort localArray ascending order by x.RRT
*X* = x.localArray(middleElement)
*Y* = y.localArray(middleElement)
Add (*X*, *Y*) at the end of newPoints vector
Increment *i* by 2
End Loop
return newPoints
**Algorithm: medianFilter5**
Input: RRT(N×2 matrix containing pairs of *x*,*y* coordinates of *N* points)
Output: newPoints (2D vector containing *x*,*y* coordinates of extracted control points)
newPoints ← initializeVector()
localArray: 5 × 2 array containing *x*,*y* coordinates to be sorted
*i* ← 2
Loop while i<N−2 do
localArray ← (RRT(i−2), RRT(i−1), RRT(*i*), RRT(i+1), RRT(i+2))
sort localArray ascending order by x.RRT
*X* = x.localArray(middleElement)
*Y* = y.localArray(middleElement)
Add (*X*, *Y*) at the end of newPoints vector
Increment *i* by 3
End Loop
return newPoints

**Table 5:** Test results for every map–algorithm pair.

Test	Algorithm	Number of Points/Nodes	Point Reduction Type	Algorithm Runtime (ms)	Travel Runtime (s) *	cmd_vel ‰ of Sharp Breaks over All Commands	Improvement in Time Compared to RRT (%)
MAP1_1	RRT	121	-	0.11	463.08	33	-
MAP1_2	BC	121	-	29.1	409.01	12	11.6
MAP1_3	BC	63/121	Mean-3	8.3	386.11	5	16.6
MAP1_4	BC	63/121	Median-3	8	387.23	7	16.3
MAP1_5	BC	42/121	Mean-5	4	381.98	5	17.5
MAP1_6	BC	42/121	Median-5	5	380.93	2	17.7
MAP2_1	RRT	74	-	0.2	287.9	41	-
MAP2_2	BC	74	-	7.3	229.2	4	20.3
MAP2_3	BC	38/74	Mean-3	2.2	216.6	13	24.7
MAP2_4	BC	38/74	Median-3	2.8	216.2	9	24.9
MAP2_5	BC	26/74	Mean-5	1.4	215.6	4	25
MAP2_6	BC	26/74	Median-5	1.5	213.1	4	25.9
MAP3_1	RRT	33	-	0.3	135.0	15	-
MAP3_2	BC	33	-	0.9	125.3	7	7.1
MAP3_3	BC	18/33	Mean-3	0.4	121.3	8	10.1
MAP3_4	BC	18/33	Median-3	0.9	121.2	8	10.2
MAP3_5	BC	12/33	Mean-5	0.5	119.4	8	11.5
MAP3_6	BC	12/33	Median-5	0.6	121.9	8	9.7
MAP4_1	RRT	118	-	0.2	571.6	37	-
MAP4_2	BC	120	-	28.6	432.5	0	24.6
MAP4_3	BC	60/118	Mean-3	8.2	377.7	7	33.9
MAP4_4	BC	60/118	Median-3	7.7	373.2	2	34.7
MAP4_5	BC	41/118	Mean-5	3.8	355.1	5	37.8
MAP4_6	BC	41/118	Median-5	4.3	352.2	2	38.3

* Travel time was calculated by taking the average of 10 travel times under the same conditions.

## Data Availability

The original contributions presented in this study are included in the article. Further inquiries can be directed to the corresponding author.

## Abbreviations

The following abbreviations are used in this manuscript:

USV	Unmanned Surface Vehicle
BC	Bézier Curve
RRT	Rapidly Exploring Random Tree
